# Supplementary material for: Treatment of Uranium-Contaminated Ground Water Using Adsorption Technology via Novel Mesoporous Silica Nanoparticles
Source: Molecules. 2023 Jul 25;28(15):5642. doi: 10.3390/molecules28155642 (PMC10420160; doi:10.3390/molecules28155642)
Supplement: Supplementary file 1 [file molecules-28-05642-s001.zip › molecules-2337688-supplementary.pdf]

molecules

MDPI

Supplementary Materials:

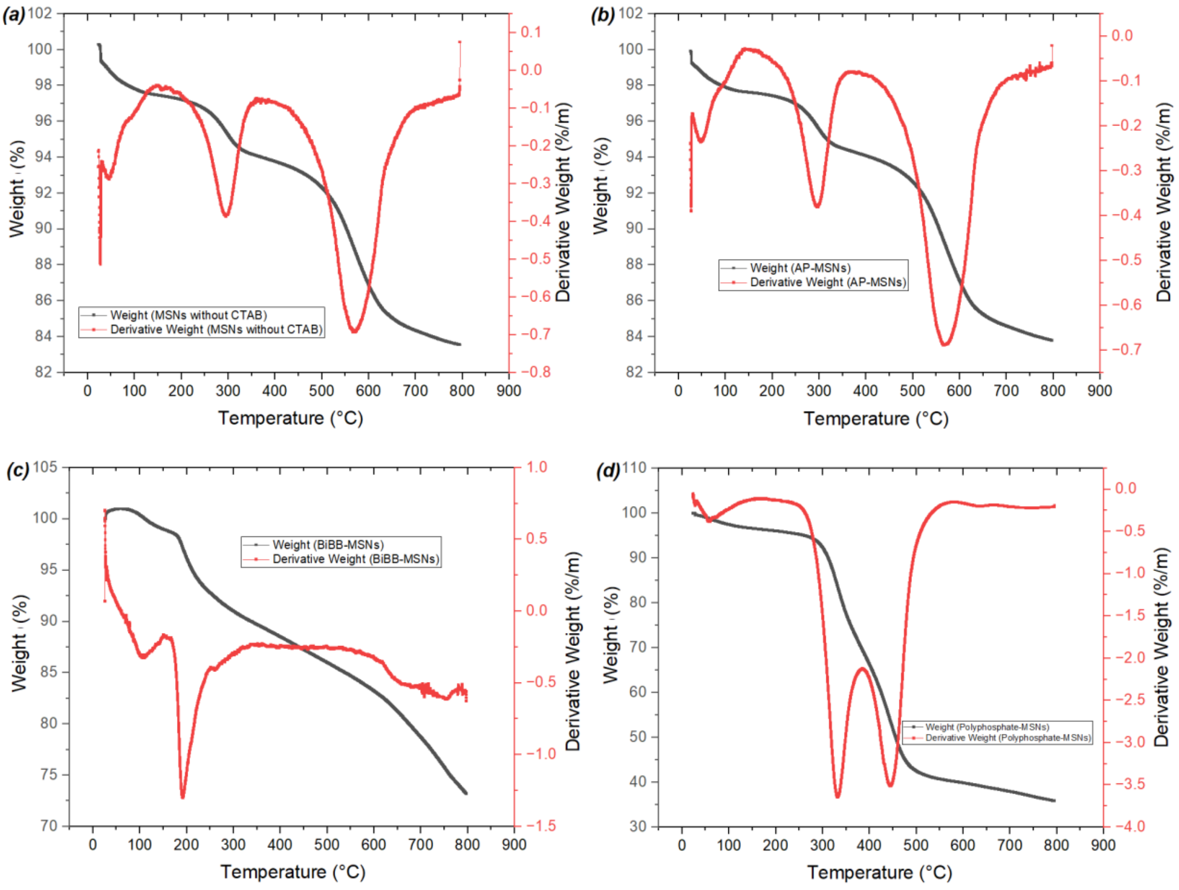

Figure 1. DTG thermal analysis curves illustrating the correlation between mass loss rate and temperature for polyphosphate-MSNs; (**a**) As made MSNs; (**b**) MSNs without CTAB; (**c**) Epoxy-MSNs; (**d**) Phosphate-MSNs.

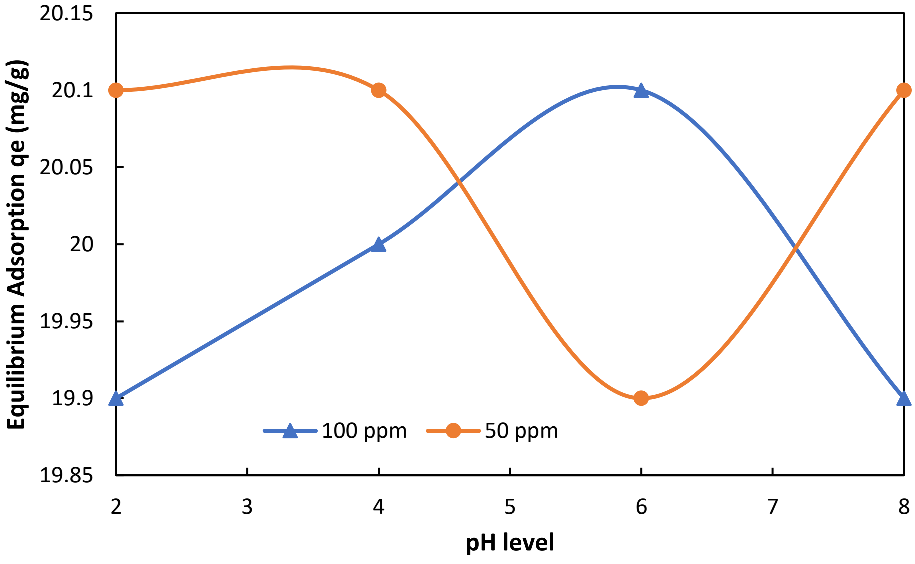

Figure 2. Equilibrium adsorption qe (mg/g) against pH levels in a Batch Adsorption Experiment.

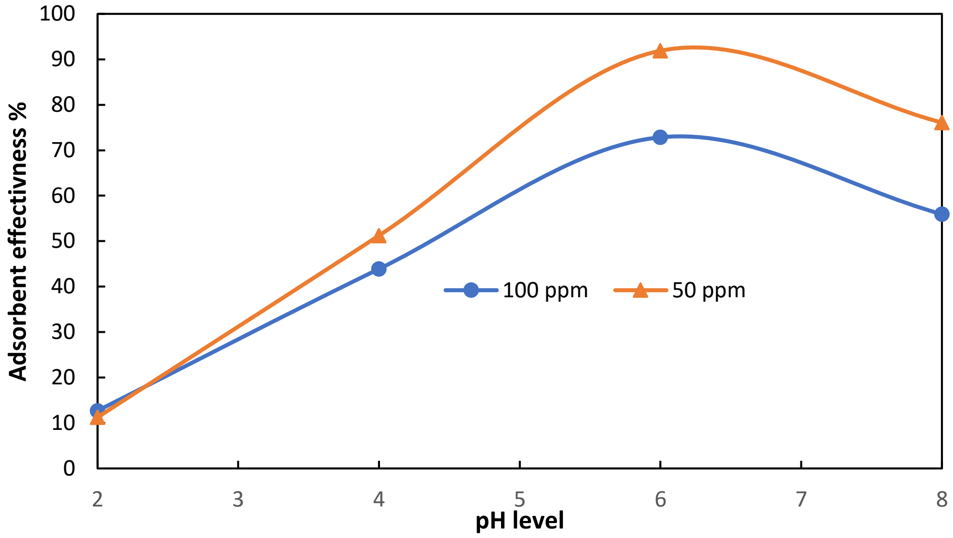

Figure 3. Adsorption effectiveness % as a function of pH level at two U concentrations (ppm).

Table 1. Effect of Time and Temperature on U Adsorption (phosphate (diphosphate)-modified MSNs (phosphate (diphosphate)-MSNs) Novel material (B):.

**A. Effect of time on U adsorption at room temperature.**				
**t (minute)**	**Ce (mg/l)**	**SD**	**%**	**qt(mg/g)**
5	75.15	1.60	24.85	19.9
10	64.6	0.98	35.40	19.9
15	64.95	0.57	35.05	19.5
30	69.71	2.30	29.59	20.4
60	68.45	1.30	30.86	20.1
120	50.37	0.30	49.12	20.1
180	55.12	1.41	44.32	20.4
240	61.60	0.94	37.78	20.3
**B. Effect of temperature on U adsorption (COF) at 25°C.**				
**U^{++} concentration (ppm)**	**Ce (mg/l)**	**SD**	**%**	**qe (mg/g)**
10	2.54	0.09	73.81	20.5
40	25.37	1.07	33.24	20.3
60	41.00	0.79	26.79	20.0
80	53.58	0.26	27.59	19.8
100	68.00	1.42	31.31	19.7
**C. Effect of temperature on U adsorption (COF) at 35°C.**				
**U^{++} concentration (ppm)**	**Ce (mg/l)**	**SD**	**%**	**qe (mg/g)**
10	4.89	0.08	49.59	20.5
40	25.60	1.43	32.63	19.6
60	41.96	1.87	25.07	20.1
80	20.00	1.20	72.97	19.5
100	25.39	1.90	74.35	19.7
**D. Effect of temperature on U adsorption (COF) at 45°C.**				
**U^{++} concentration (ppm)**	**Ce (mg/l)**	**SD**	**%**	**qe (mg/g)**
10	1.21	0.08	87.53	19.8
40	22.16	0.86	41.68	19.5
60	33.88	1.43	39.50	20.2
80	53.46	2.72	27.76	19.7

100	62.42	1.32	36.95	19.6
**E. Effect of temperature on U adsorption (COF) at 55°C.**				
**U⁺⁺ concentration (ppm)**	**Ce (mg/l)**	**SD**	**%**	**qe (mg/g)**
10	1.29	0.02	86.70	19.6
40	22.57	0.62	40.61	20.0
60	36.00	0.84	35.71	20.2
80	19.17	0.43	74.09	19.7
100	52.50	0.78	46.97	19.8

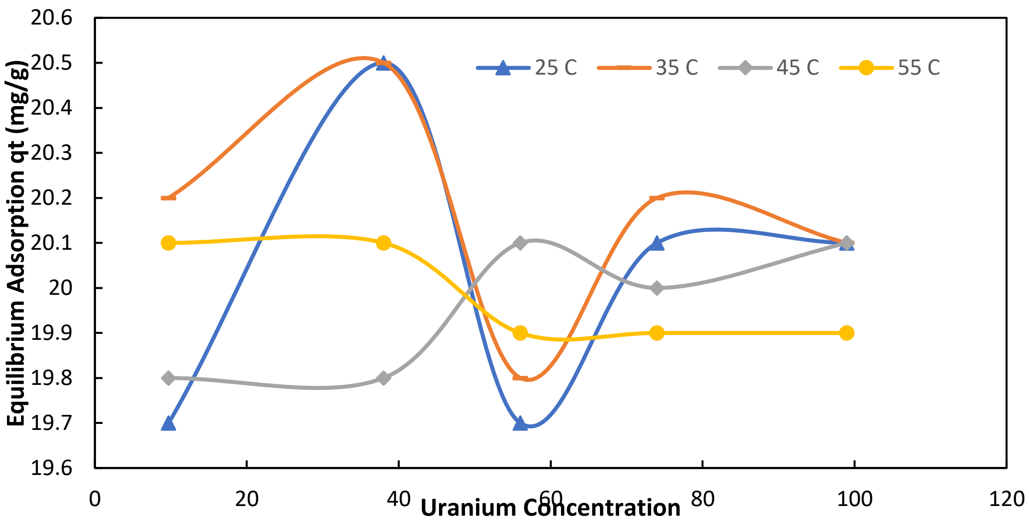

Figure 4. Equilibrium adsorption qe (mg/g) against U concentration (µg/L) in a Batch Adsorption Experiment.

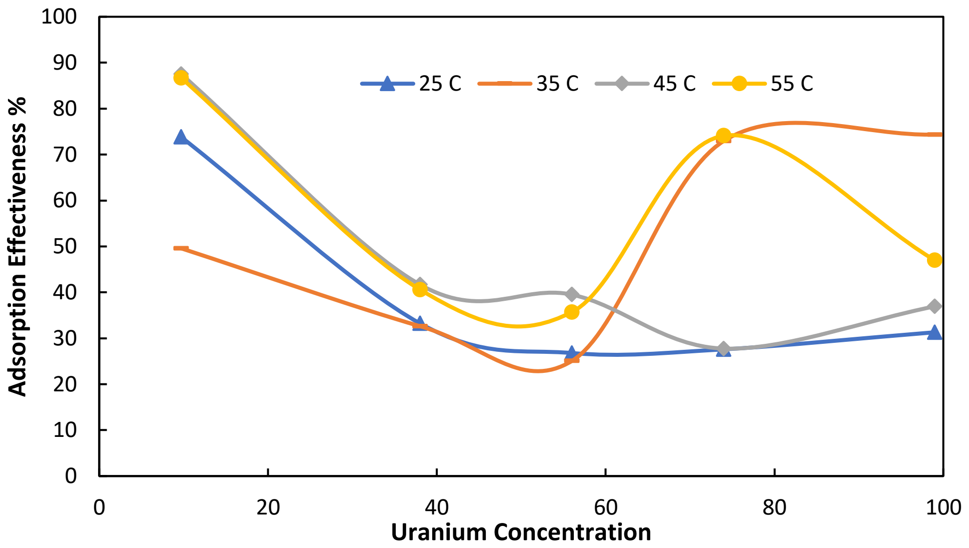

Figure 5. Adsorption effectiveness (%) against U concentration for synthesized materials.

Table 2. Effect of pH on Adsorption at Two U concentrations (50 and 100 ppm) at Room Temperature (phosphate (diphosphate)-modified MSNs (phosphate (diphosphate)-MSNs) Novel material (B)).

**A. 60 minutes at U concentration 100 ppm.**					
**pH**	**Ci (mg/l)**	**Ce (mg/l)**	**SD**	**%**	**qe (mg/g)**
2	100	94.64	0.51	5.36	19.8
4	100	76.28	1.20	23.72	19.6
6	100	68.45	1.56	31.55	20.0
8	100	82.86	0.29	17.14	19.8
**B. 60 minutes at U concentration 50 ppm.**					
**pH**	**Ci (mg/l)**	**Ce (mg/l)**	**SD**	**%**	**qe (mg/g)**
2	50	48.00	1.02	4.00	19.5
4	50	31.95	0.33	36.10	19.7
6	50	37.58	1.44	24.84	20.4
8	50	40.55	0.95	18.90	20.1

Table 3. Effect of material weight at room temperature for 60 minutes (U concentration 50, 100 ppm) (phosphate (diphosphate)-modified MSNs (phosphate (diphosphate)-MSNs) Novel material (B)).

**A. 60 minutes at U concentration 100 ppm.**				
**Weight (mg)**	**Ce (mg/l)**	**SD**	**%**	**qe (mg/g)**
10	63.50	1.05	36.50	9.6
15	66.88	1.51	33.12	14.8
20	68.45	0.86	31.55	20.1
25	64.45	0.30	35.36	24.7
30	61.56	1.45	38.44	29.7
50	65.57	0.67	34.43	49.9
**B. 60 minutes at U concentration 50 ppm.**				
**Weight (mg)**	**Ce (mg/l)**		**%**	**qe (mg/g)**
10	9.60	0.53	36.34	9.8
15	14.80	1.10	35.00	15.1
20	20.10	1.40	35.46	20.1
25	24.70	0.96	38.54	24.6
30	29.70	0.43	39.74	30.3
50	49.90	2.33	21.54	50.1

Table 4. Effect of mixture solution at room temperature for 60 minutes (phosphate (diphosphate)-modified MSNs (phosphate (diphosphate)-MSNs) Novel material (B)).

**A. The effect of adsorption with Pb.**				
**Element**	**Ce (mg/l)**	**SD**	**%**	**qe (mg/g)**
Cr	82	0.49	18	
Ni	84	1.05	16	
Cu	91	1.45	9	
Zn	89	1.09	11	50
Cd	90	1.17	10	
Pb	84	0.63	16	
U	60	0.62	40	
**B. The effect of adsorption in the absence of Pb.**				
**Element**	**Ce (mg/l)**	**SD**	**%**	**qe (mg/g)**
Cr	84	1.33	16	
Ni	81	0.82	19	
Cu	90	0.83	10	49.7
Zn	88	1.04	12	
Cd	95	1.18	5	
U	63	0.89	37	

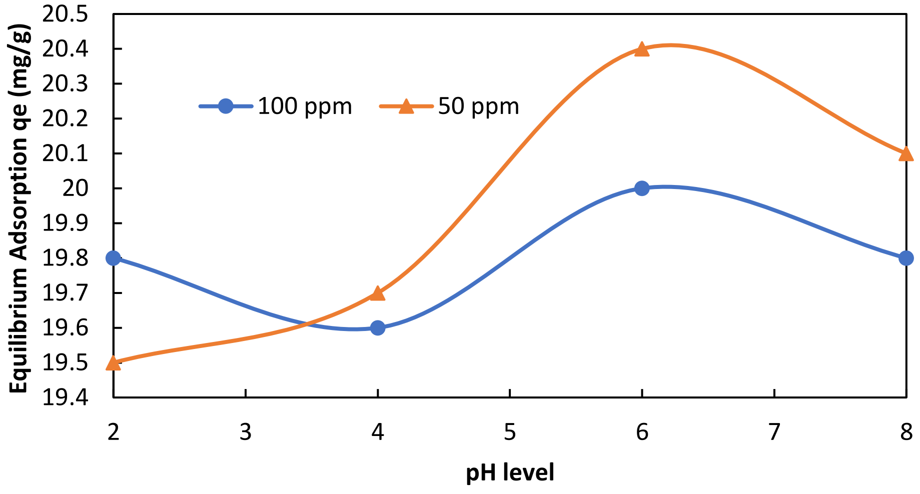

Figure 6. Equilibrium adsorption qe (mg/g) against pH levels in a Batch Adsorption Experiment.

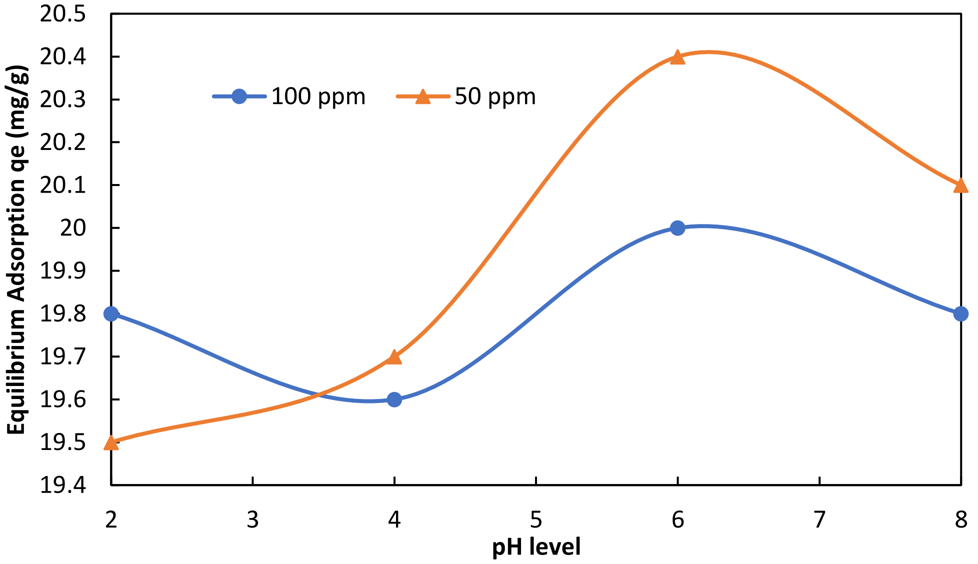

Figure 7. Adsorption Effectiveness (%) as a function of pH levels in a Batch Adsorption Experiment.
